# Supplementary material for: Functional neuronal circuits emerge in the absence of developmental activity
Source: Nat Commun. 2024 Jan 8;15:364. doi: 10.1038/s41467-023-44681-2 (PMC10774424; doi:10.1038/s41467-023-44681-2)
Supplement: Supplementary file 3 — Description of Additional Supplementary Files [file 41467_2023_44681_MOESM3_ESM.pdf]

<u>Description of Additional Supplementary Files</u>

Supplementary Movies

Supplementary Movie 1 We illustrate the on-rate of tricaine by applying anesthesia to a fish reared under standard conditions. We pour the anesthesia on at 10s, and for the following 20s the fish slows its swim bouts. After 30-40s, the fish floats, losing posture and no longer responding to physical stimuli.

Supplementary Movie 2 We illustrate the off-rate of tricaine by washing out the anesthesia in the fish in Supplemental Movie 1. In the first 10s, the fish is in a tricaine bath, and continues to be immobile and does not respond to physical touch. We then transfer the fish to a bath of standard fish water by 20s, and after 1 minute and 10 seconds we see the first touch-evoked swim. The fish continues to recover for the next minute, and by 2 minutes after wash out begins to swim in responses to taps.

Supplementary Movie 3 Full anatomy stacks of four normally reared (left) and tricaine-reared (right) fish shown in Supplemental Figure 10.

Supplementary Movie 4 Recording of fish in Figure 4 while still under tricaine (first 303 frames, 10s at 30 frames/s) and after washout (frames 304-1201, last 30s at 30 frames/s). No coordinated activity is observed while still under anesthesia, despite being shown continuous visual stimuli, while strong coordinated activity is seen even immediately after washout.
