# Supplementary material for: Functional neuronal circuits emerge in the absence of developmental activity
Source: Nat Commun. 2024 Jan 8;15:364. doi: 10.1038/s41467-023-44681-2 (PMC10774424; doi:10.1038/s41467-023-44681-2)
Supplement: Supplementary file 8 — Reporting Summary [file 41467_2023_44681_MOESM8_ESM.pdf]

nature portfolio

Corresponding author(s): *Double-anonymous peer review submissions: write DAPR and your manuscript number here instead of author names.*

Last updated by author(s): *YYYY-MM-DD*

Reporting Summary

Nature Portfolio wishes to improve the reproducibility of the work that we publish. This form provides structure for consistency and transparency in reporting. For further information on Nature Portfolio policies, see our Editorial Policies and the Editorial Policy Checklist.

Statistics

For all statistical analyses, confirm that the following items are present in the figure legend, table legend, main text, or Methods section.

n/a	Confirmed	
☐	☒	The exact sample size (*n*) for each experimental group/condition, given as a discrete number and unit of measurement
☐	☒	A statement on whether measurements were taken from distinct samples or whether the same sample was measured repeatedly
☐	☒	The statistical test(s) used AND whether they are one- or two-sided<br>*Only common tests should be described solely by name; describe more complex techniques in the Methods section.*
☒	☐	A description of all covariates tested
☒	☐	A description of any assumptions or corrections, such as tests of normality and adjustment for multiple comparisons
☐	☒	A full description of the statistical parameters including central tendency (e.g. means) or other basic estimates (e.g. regression coefficient) AND variation (e.g. standard deviation) or associated estimates of uncertainty (e.g. confidence intervals)
☐	☒	For null hypothesis testing, the test statistic (e.g. *F*, *t*, *r*) with confidence intervals, effect sizes, degrees of freedom and *P* value noted<br>*Give P values as exact values whenever suitable.*
☒	☐	For Bayesian analysis, information on the choice of priors and Markov chain Monte Carlo settings
☒	☐	For hierarchical and complex designs, identification of the appropriate level for tests and full reporting of outcomes
☒	☐	Estimates of effect sizes (e.g. Cohen's *d*, Pearson's *r*), indicating how they were calculated

Our web collection on statistics for biologists contains articles on many of the points above.

Software and code

Policy information about availability of computer code

Data collection	Data collection was done using the code and methods described in https://doi.org/10.1038/s41593-019-0534-9
Data analysis	All data analysis was performed in Python 3 using custom written code, available at https://github.com/bdanubius/InnateFish. For analyzing 2P imaging stacks, we used the open-source CalmAn framework (https://github.com/flatironinstitute/CalmAn) for motion alignment, segmentation, and signal extraction. For mapping imaging volume to the z-brain reference, we used the open-source Computational Morphometry Toolkit (CMTK 3.3.1) and the open-source Advanced Normalization Tools (ANTs 2.3.1).

For manuscripts utilizing custom algorithms or software that are central to the research but not yet described in published literature, software must be made available to editors and reviewers. We strongly encourage code deposition in a community repository (e.g. GitHub). See the Nature Portfolio guidelines for submitting code & software for further information.

Data

Policy information about availability of data

All manuscripts must include a data availability statement. This statement should provide the following information, where applicable:

- Accession codes, unique identifiers, or web links for publicly available datasets
- A description of any restrictions on data availability
- For clinical datasets or third party data, please ensure that the statement adheres to our policy

Source data are provided with this paper. The raw imaging data have file sizes on the order of terabytes, and can be shipped on a hard drive at request from the corresponding author.

Research involving human participants, their data, or biological material

Policy information about studies with human participants or human data. See also policy information about sex, gender (identity/presentation), and sexual orientation and race, ethnicity and racism.

Reporting on sex and gender	n/a
Reporting on race, ethnicity, or other socially relevant groupings	n/a
Population characteristics	n/a
Recruitment	n/a
Ethics oversight	n/a

Note that full information on the approval of the study protocol must also be provided in the manuscript.

Field-specific reporting

Please select the one below that is the best fit for your research. If you are not sure, read the appropriate sections before making your selection.

☒ Life sciences ☐ Behavioural & social sciences ☐ Ecological, evolutionary & environmental sciences

For a reference copy of the document with all sections, see nature.com/documents/nr-reporting-summary-flat.pdf

Life sciences study design

All studies must disclose on these points even when the disclosure is negative.

Sample size	For all studies quantifying OMR behavior we ran at least N = 64 fish, significantly above the N = 30 recommended by papers that utilized the same behavioral rig (https://doi.org/10.1038/s41593-019-0534-9), although exclusions applied that lowered these numbers in certain conditions. For imaging, we ran at least 7 fish per condition, and utilized the three of the brightest samples per condition. For the embedded imaging experiments (Fig. 2, Fig. 4), variability between fish was low, and hence, we planned experiments for around N = 3 fish. We then performed experiments in batches according to these numbers.
Data exclusions	For freely swimming fish, we excluded animals when they did not swim at all or spent most of the time near the wall of the experimental chamber, and therefore could not be tracked. For anesthesia-reared fish, we discarded all fish in a petri dish if we found any animal that responded to visual or physical stimulus. We excluded any day of behavioral recording when the wild type (untreated fish) showed significant deviations from normal behavior. We excluded fish with weak GCaMP activity from neural recordings.
Replication	Data for each condition was collected on separate days and over multiple months, and from multiple parent batches, all with consistent results. For behavioral data, this represented daily testing of WT fish, and at least a few days of repeats of all other treatments. 2-photon imaging studies were performed over the course of 2 months, providing 3 replicates per control and treated condition, with additional replicates eliminated when GCaMP activity was low.
Randomization	Fish were randomly assigned to treated, untreated, and recovery conditions. The order of visual stimuli was always presented randomly.
Blinding	All data analysis was automatic, and hence blind, and was not different between different conditions within one experiment. Investigators were not blinded to group allocation during data collection, however, most, if not all, data collection occurred using automated behavioral experiments.

Reporting for specific materials, systems and methods

We require information from authors about some types of materials, experimental systems and methods used in many studies. Here, indicate whether each material, system or method listed is relevant to your study. If you are not sure if a list item applies to your research, read the appropriate section before selecting a response.

Materials & experimental systems

n/a	Involved in the study
☒	☐ Antibodies
☒	☐ Eukaryotic cell lines
☒	☐ Palaeontology and archaeology
☐	☒ Animals and other organisms
☒	☐ Clinical data
☒	☐ Dual use research of concern
☒	☐ Plants

Methods

n/a	Involved in the study
☒	☐ ChIP-seq
☒	☐ Flow cytometry
☒	☐ MRI-based neuroimaging

Animals and other research organisms

Policy information about studies involving animals; ARRIVE guidelines recommended for reporting animal research, and Sex and Gender in Research

Laboratory animals	WIK11 (Wild-caught Indian Zebrafish), elavl3:GCaMP6s zebrafish. All fish were analyzed at 0-6 dpf.
Wild animals	No wild animals were used in this study.
Reporting on sex	No selection for sex was made for the fish utilized in this study, thus we assume an even gender split. The first indication of zebrafish sex differentiation is known to be at 10-12 dpf, past the 6 dpf experiments we utilize.
Field-collected samples	No field-collected samples were used in this study.
Ethics oversight	All experiments were approved by the Harvard University standing committee on the use of animals in research and training.

Note that full information on the approval of the study protocol must also be provided in the manuscript.

Plants

Seed stocks	No plants were used in this study.
Novel plant genotypes	No plants were used in this study.
Authentication	No plants were used in this study.
