# Supplementary material for: Functional neuronal circuits emerge in the absence of developmental activity
Source: Nat Commun. 2024 Jan 8;15:364. doi: 10.1038/s41467-023-44681-2 (PMC10774424; doi:10.1038/s41467-023-44681-2)

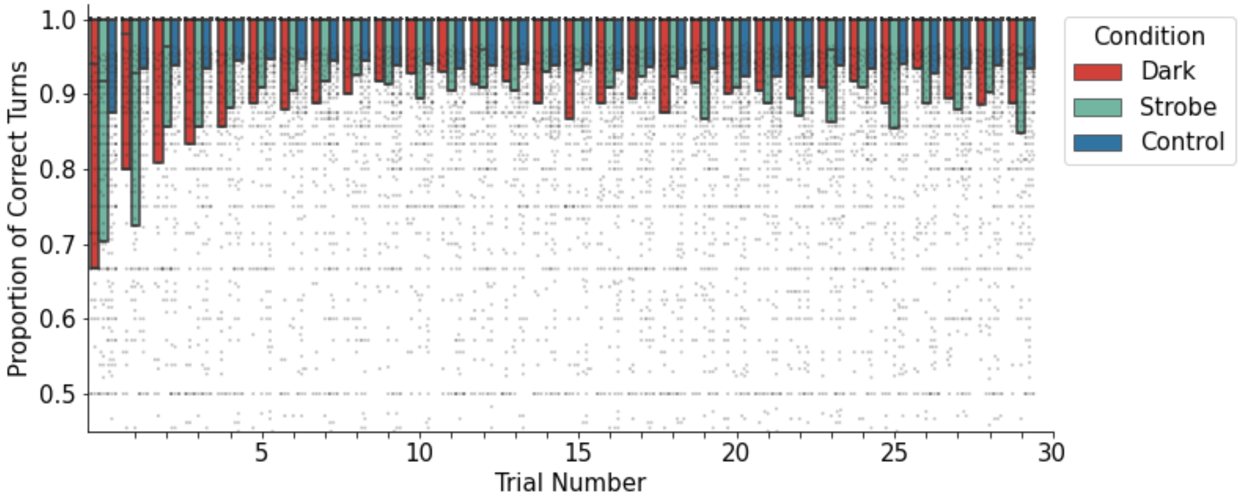

Supplemental Figure 1: Fish performance over multiple trials in a behavioral session.
Proportion of correct turns made by control fish, dark-reared fish, and strobe-reared fish as a function of trial numbers during a behavior session. Bars show quartile range and median for each condition. n: Control: 392 fish; Dark: 102; Strobe: 95.

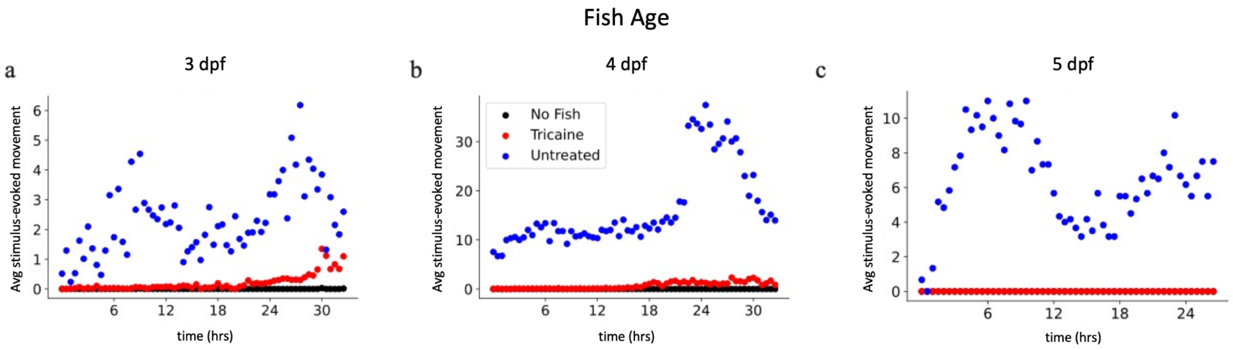

Supplemental Figure 2: Half-life of continual anesthesia application.
(a-c) Quantification of the half-life of anesthesia treatment at various days of maturation. Fish are placed in 96 well plates in the dark at 3 days (a), 4 days (b) and 5 days (c) of age, prior to which they are kept in anesthesia. After being placed in the 96 well plates, anesthesia is not replaced, thus time on the x-axis indicates hours since tricaine was last applied. During the experiment, fish are shown a 30s light on and then a 30s light off stimulus every 10 minutes, and we record resultant motion. In all three cases we see clear motion in untreated fish throughout the experiments, while we see little to no motion in fish raised in tricaine prior to 18 hours of treatment. For this reason, we exchange the tricaine bath of fish every 12 hours, in order to confirm complete anesthetization during development. In the rare cases motion is observed in the tricained fish (~4 hours on Day 3 or ~ 9 hours on Day 5), we also observed motion in the wells in which we did not place fish, indicating that we are experiencing either shot noise or motion in the rig room, rather than observing tricainated fish that have "woken up".

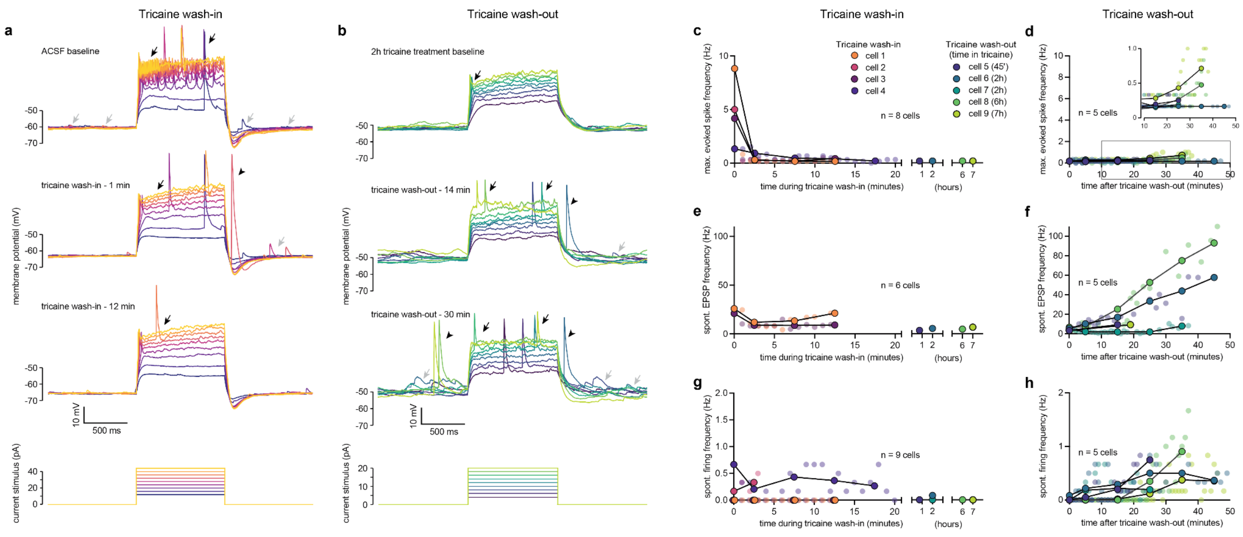

Supplemental Figure 3: *In-vivo* whole-cell electrophysiology of putative Purkinje cells during acute tricaine wash-in and wash-out following extended durations of tricaine treatment.

a) Example traces showing cellular responses of a putative Purkinje cell to somatic current injections (bottom) during baseline recording in ACSF (top) and after 1 minute and 12 minutes during acute wash-in of tricaine-ACSF.

b) Example traces showing cellular responses of a putative Purkinje cell to somatic current injections (bottom) after the animal was kept in tricaine-ACSF for 2 hours prior to recording and after 14 minutes and 30 minutes during tricaine wash-out.

a, b) Black arrows, evoked spikes, arrowheads, spontaneous spikes; grey arrows, EPSPs.

c) Scatter plot showing the maximum spike rate that could be evoked during periods of current stimulation for different durations of tricaine exposure. Connected dots, averaged data in 10 minute bins.

d) Scatter plot showing the maximum spike rate that could be evoked during periods of current stimulation during tricaine wash-out in fish that had been anesthetized for 45 minutes to 7 hours.

e) Scatter plot showing the rate of spontaneous EPSPs occurring during periods of no current stimulation for different durations of tricaine exposure. Connected dots, averaged data in 10 minute bins.

f) Scatter plot showing the rate of spontaneous EPSPs occurring during periods of no current stimulation during tricaine wash-out in fish that had been anesthetized for 45 minutes to 7 hours.

g) Scatter plot showing the rate of spontaneous spikes occurring during periods of no current stimulation for different durations of tricaine exposure. Connected dots, averaged data in 10 minute bins.

h) Scatter plot showing the rate of spontaneous spikes occurring during periods of no current stimulation during tricaine wash-out in fish that had been anesthetized for 45 minutes to 7 hours.

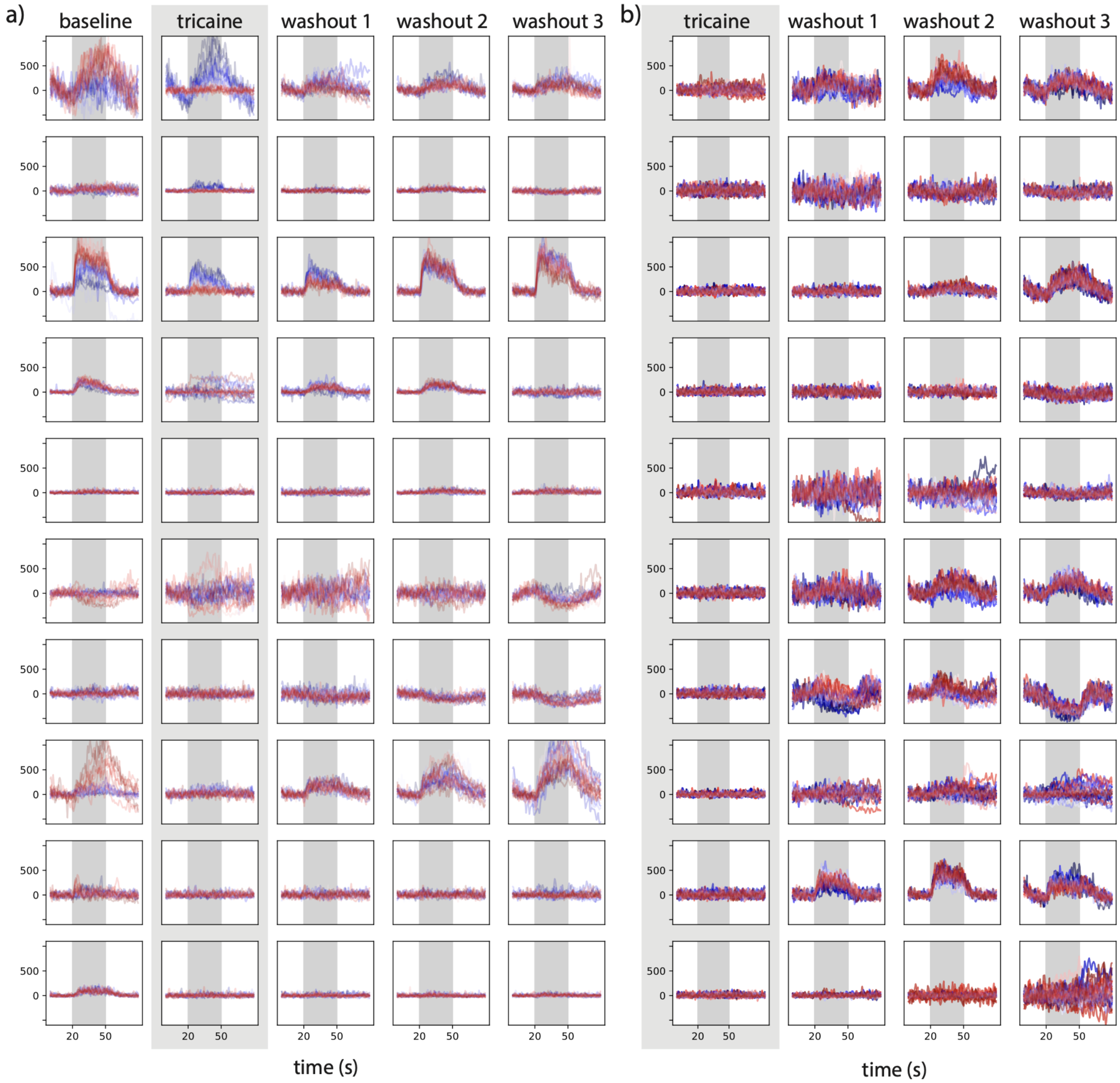

Supplemental Figure 4: Most active units during tricaine application.
Plots showing the ten units that were most active during the tricaine period for both short-term anesthesia (a) and lifetime anesthesia exposure (b), with the top 3 most active cells marked in Figures 2d and 4d. Y-axis plots responsiveness index ($C-C_0$, see Methods). Each row is a single unit over multiple hours of imaging, and each plot shows all trials of the preferred direction during that imaging block. Trials are color-coded by order in the imaging session, with blue indicating early trials and red corresponding to trials later in the imaging session (see color bar right of b). Note that units experiencing temporary anesthesia may exhibit activity early in the imaging session, but steadily ramp down in activity by the end of the tricaine period. By contrast, the most active cells in animals raised under anesthesia mostly correspond to noisy cells with weak activity even during washout, highlighting the effects of normalization in Figures 2d,i and 4d,i.

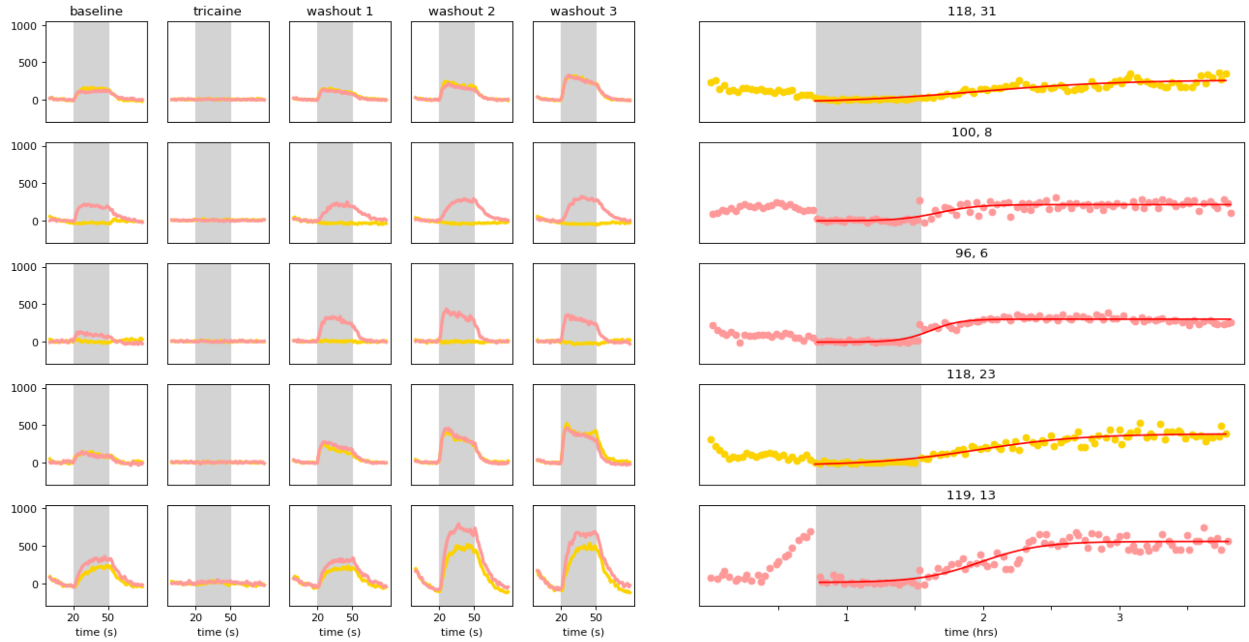

Supplemental Figure 5: Units that become active in the first hour after short-term anesthesia washout.

Example units and fits from fish exposed to short-term anesthesia. Y-axis plots responsiveness index ($C\text{-}C_o$, see Methods). Square plots show the average response of the unit to leftward (pink) and rightward (yellow) motion averaged over an entire imaging session (akin to Figure 2f-h). Scatter plots show responsiveness-index of the preferred direction (indicated by color) of units in tectum, pretectum, and hindbrain (see Methods, Figure 2d, 4d), as well as a logistic fit to recovery. Scatter plots are titled by the midpoint (x_o) and time constant (k) of the logistic fit (plotted in Figure 4k-l).

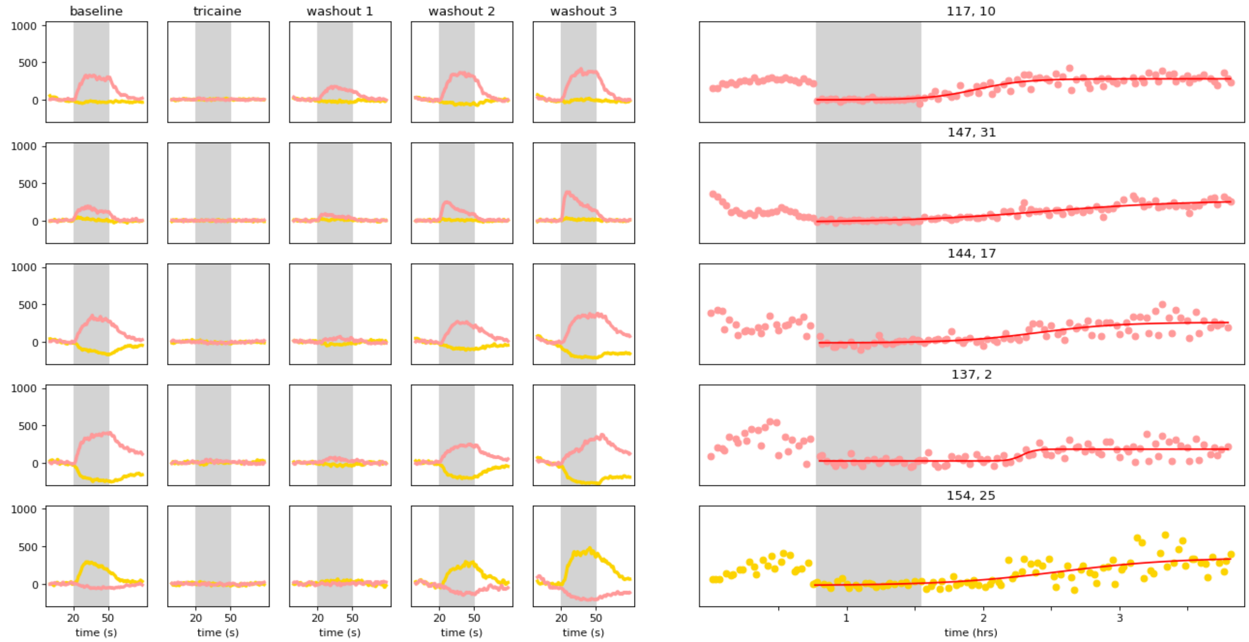

Supplemental Figure 6: Units that become active in the second hour after short-term anesthesia washout.

Example units and fits from fish exposed to short-term anesthesia. Y-axis plots responsiveness index ($C-C_o$, see Methods). Square plots show the average response of the unit to leftward (pink) and rightward (yellow) motion averaged over an entire imaging session (akin to Figure 2f-h). Scatter plots show responsiveness-index of the preferred direction (indicated by color) of units in tectum, pretectum, and hindbrain (see Methods, Figure 2d, 4d), as well as a logistic fit to recovery. Scatter plots are titled by the midpoint (x_o) and time constant (k) of the logistic fit (plotted in Figure 4k-l).

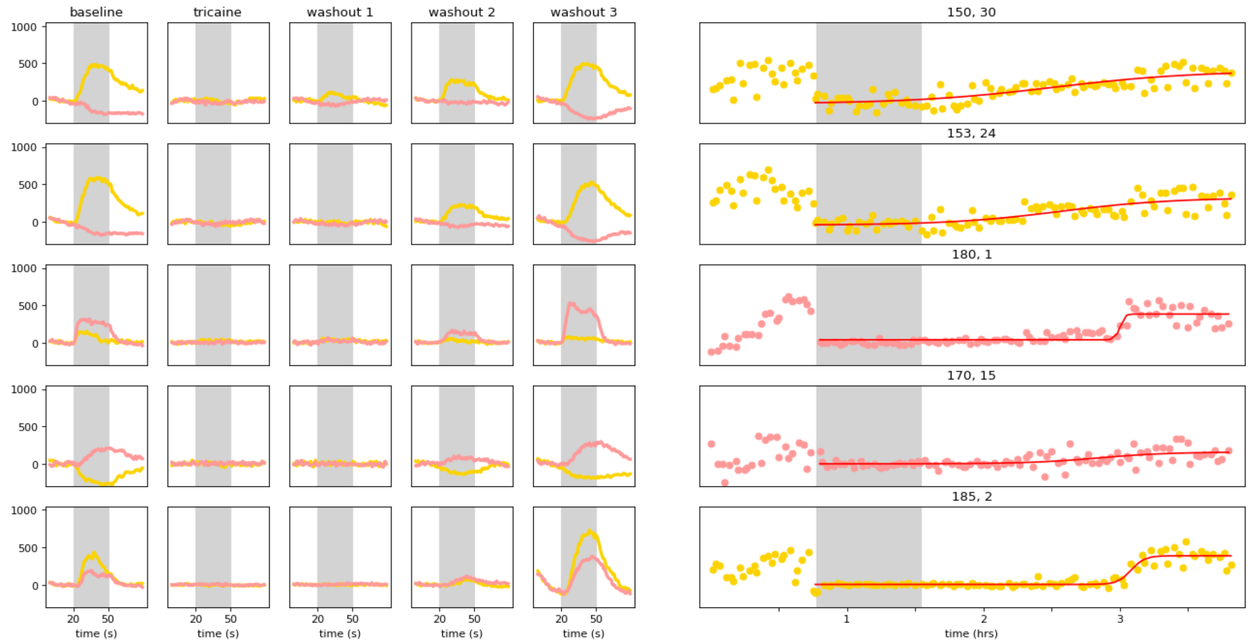

Supplemental Figure 7: Units that become active in the third hour after short-term anesthesia washout.

Example units and fits from fish exposed to short-term anesthesia. Y-axis plots responsiveness index ($C-C_o$, see Methods). Square plots show the average response of the unit to leftward (pink) and rightward (yellow) motion averaged over an entire imaging session (akin to Figure 2f-h). Scatter plots show responsiveness-index of the preferred direction (indicated by color) of units in tectum, pretectum, and hindbrain (see Methods, Figure 2d, 4d), as well as a logistic fit to recovery. Scatter plots are titled by the midpoint (x_o) and time constant (k) of the logistic fit (plotted in Figure 4k-l).

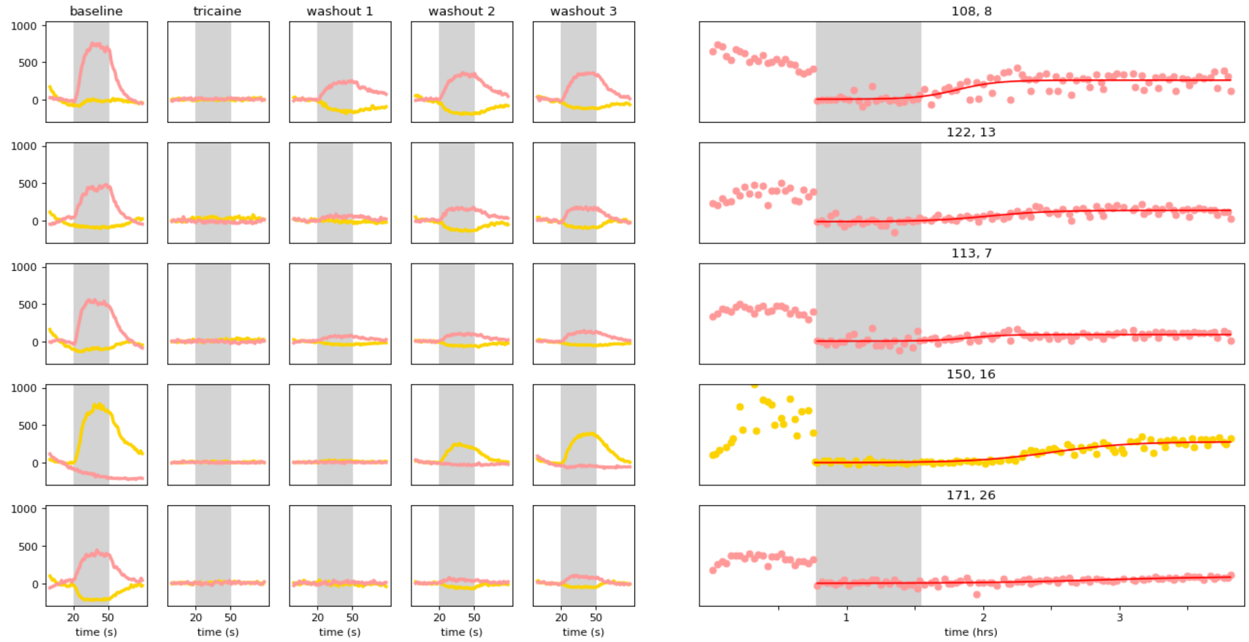

Supplemental Figure 8: Units that only weakly recover after short-term anesthesia washout.
Example units and fits from fish exposed to short-term anesthesia. Y-axis plots responsiveness index (C-C_o, see Methods). Square plots show the average response of the unit to leftward (pink) and rightward (yellow) motion averaged over an entire imaging session (akin to Figure 2f-h). Scatter plots show responsiveness-index of the preferred direction (indicated by color) of units in tectum, pretectum, and hindbrain (see Methods, Figure 2d, 4d), as well as a logistic fit to recovery. Scatter plots are titled by the midpoint (x_o) and time constant (k) of the logistic fit (plotted in Figure 4k-l).

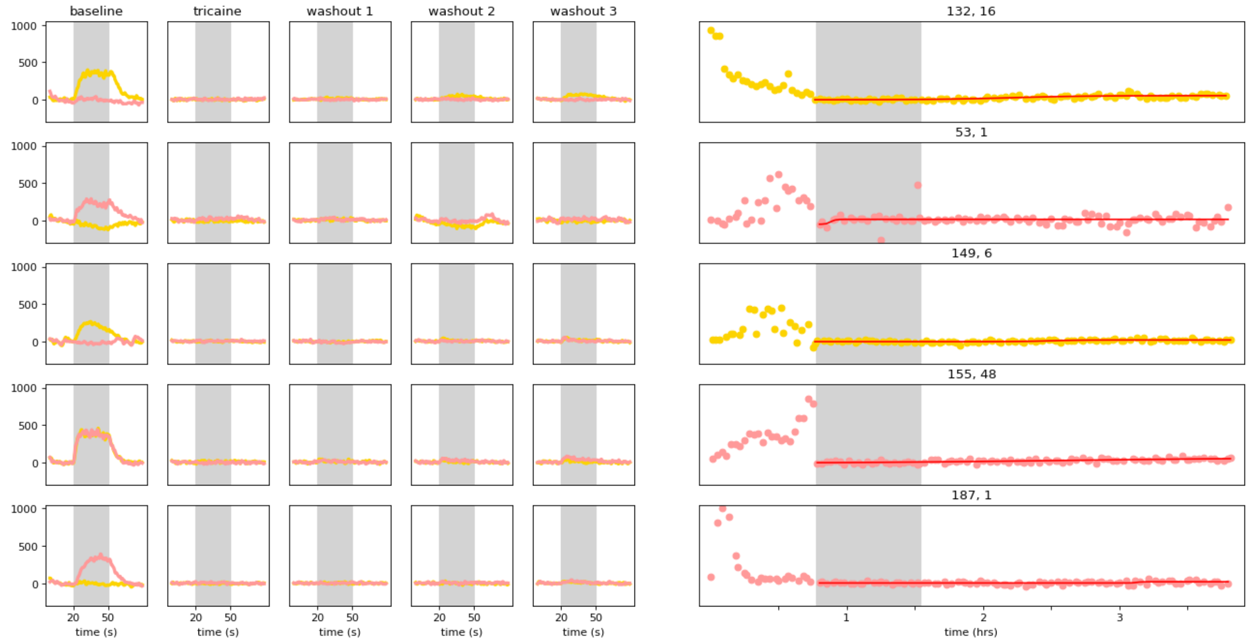

Supplemental Figure 9: Units that do not recover after short-term anesthesia washout.
Example units and fits from fish exposed to short-term anesthesia. Y-axis plots responsiveness index ($C-C_0$, see Methods). Square plots show the average response of the unit to leftward (pink) and rightward (yellow) motion averaged over an entire imaging session (akin to Figure 2f-h). Scatter plots show responsiveness-index of the preferred direction (indicated by color) of units in tectum, pretectum, and hindbrain (see Methods, Figure 2d, 4d), as well as a logistic fit to recovery. Scatter plots are titled by the midpoint (x_0) and time constant (k) of the logistic fit (plotted in Figure 4k-l).

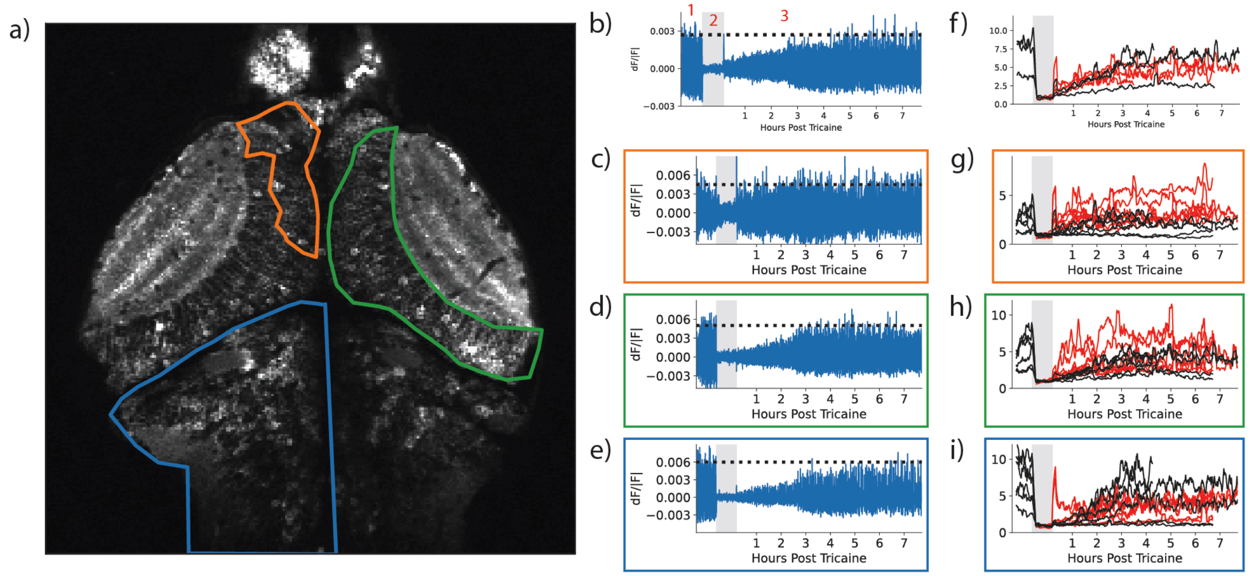

Supplemental Figure 10: Effect of tricaine anesthesia on regional activity.
(a) Average image of neural activity recording prior to tricaine administration of fish in Figure 2. Highlighted is the left hindbrain (blue), right tectal cells (green) and left pretectum (orange).
(b) Change in fluorescence (dF/|F|) of the whole imaging plane (1) during baseline, (2) during tricaine administration (2, gray region), and (3) after tricaine washout for the fish in (a). Dashed line indicates average activity before tricaine administration. Application of anesthesia leads to a categorical decrease in evoked activity. Activity increases immediately after washout, but takes 4-6 hours for a full recovery to baseline levels.
(c-e) Evoked activity (dF/|F|) of (c) pretectum, (d) tectum, and (e) hindbrain before, during and after tricaine application. All show a significant decrease in activity under tricaine application, but have different rates of recovery: pretectum (c) recovers to baseline immediately after washout in this fish, tectum (d) takes up to 3 hours for a full recovery, and hindbrain (e) can take up to 7 hours to recover to baseline levels.
(f-h) Evoked activity for multiple fish of the entire imaging plane (f), pretectum (g), tectum (h), and hindbrain (i). Black traces correspond to fish raised normally and placed under tricaine for 1 hour, while red traces indicate fish that were raised in

anesthesia and had tricaine washed out for the first time during imaging. The timescales of recovery seem overall identical independent of the length of anesthetic application, supporting the results of Figures 3 and 4. Over all fish activity recovers rapidly in the first 2-3 hours of washout, then seems to gradually to a maximum level by 6 hours, supporting the 0, 2 and 6 hour timepoints of behavioral testing in Figure 3. In these subpanels we are plotting the upper envelope of the dF/|F| shown in (b-e), normalized to the tricaine period.

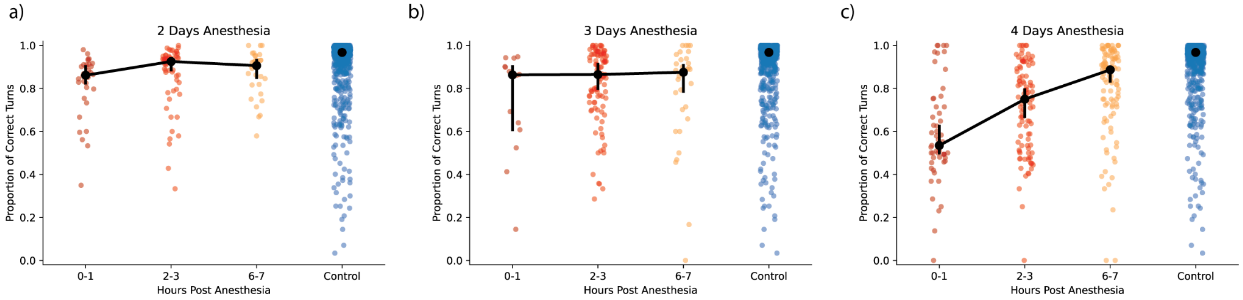

Supplemental Figure 11: OMR Performance under varying days of anesthesia application.

Proportion of correct turns during OMR made by control fish (blue, right) or fish raised under anesthesia from for 2 (a), 3 (b) or 4 (c) days of anesthesia. In all cases, the tricaine exposure included the days immediately preceding testing (days 4-6, 3-6, and 2-6, respectively). Groups were tested after 0, 2 or 6 hours of recovery after washout (red, orange, yellow, respectively). Colored circles, individual fish's performance over all trials; black circles, median of correct turns of all fish within a condition; error bars: quartile range.

n-values:

Control: 790

(a) 0-1: 32; 2-3: 59; 6-7: 30

(b) 0-1: 14; 2-3: 101; 6-7: 32

(c) 0-1: 54; 2-3: 98; 6-7: 104

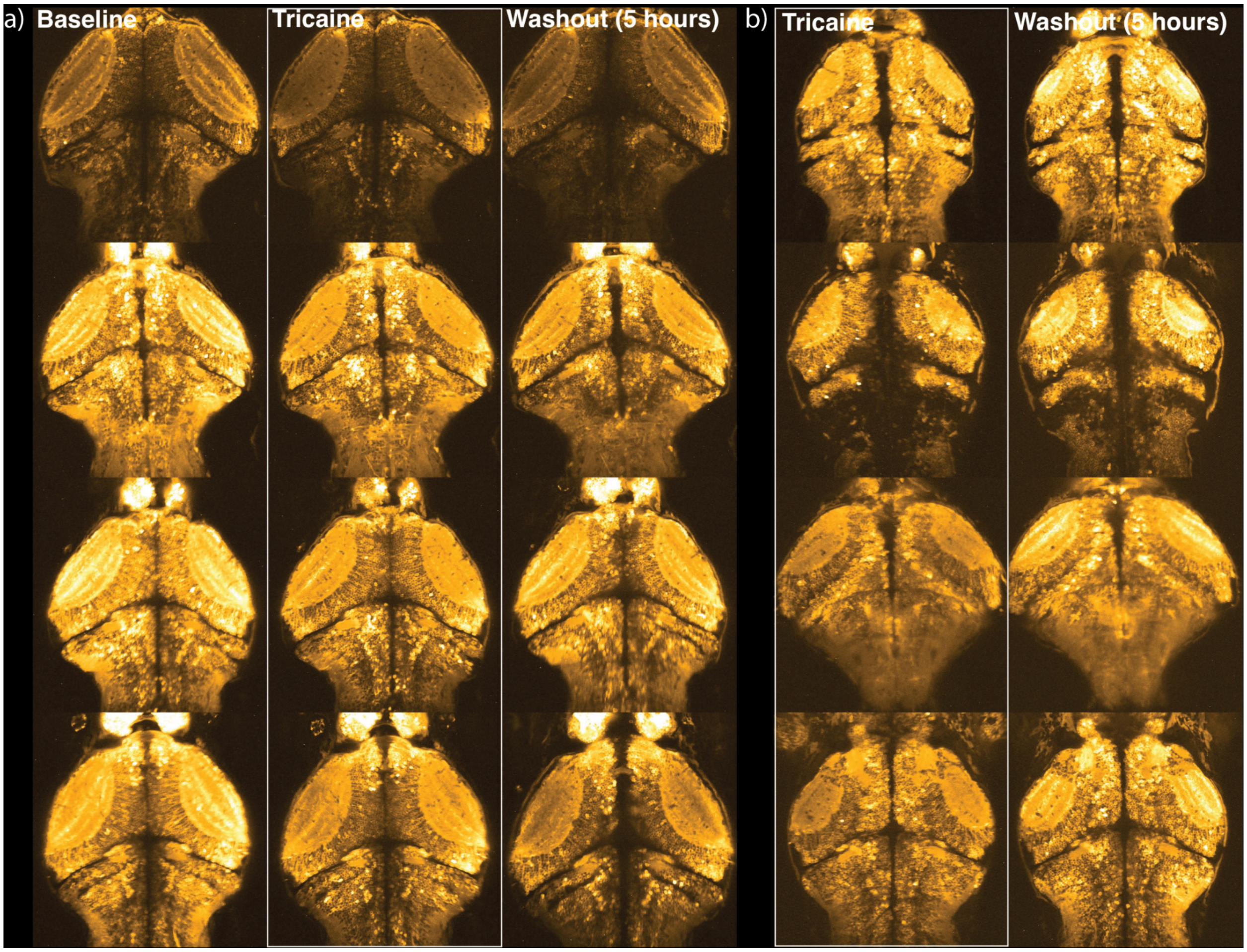

Supplemental Figure 12: Anatomical Comparison of normally- and tricaine-reared fish.

Averaged calcium signal in four normally-reared fish (a) and in a tricaine-reared fish (b) during anesthesia and after 5 hours of washout. Baselines (pre-anesthesia application) are also included for normally-reared fish. The tricaine period appears bright as we re-normalized the image to allow for the anatomical quantification in Figure 4j, rather than aiming to provide a representative view of the effect of anesthesia on fluorescence. See Figures 2d,i and 4d,i and Supplemental Figure 8 for evidence that neural activity is eliminated during tricaine application. Additional anatomy stacks are provided in Supplemental Movie 3.

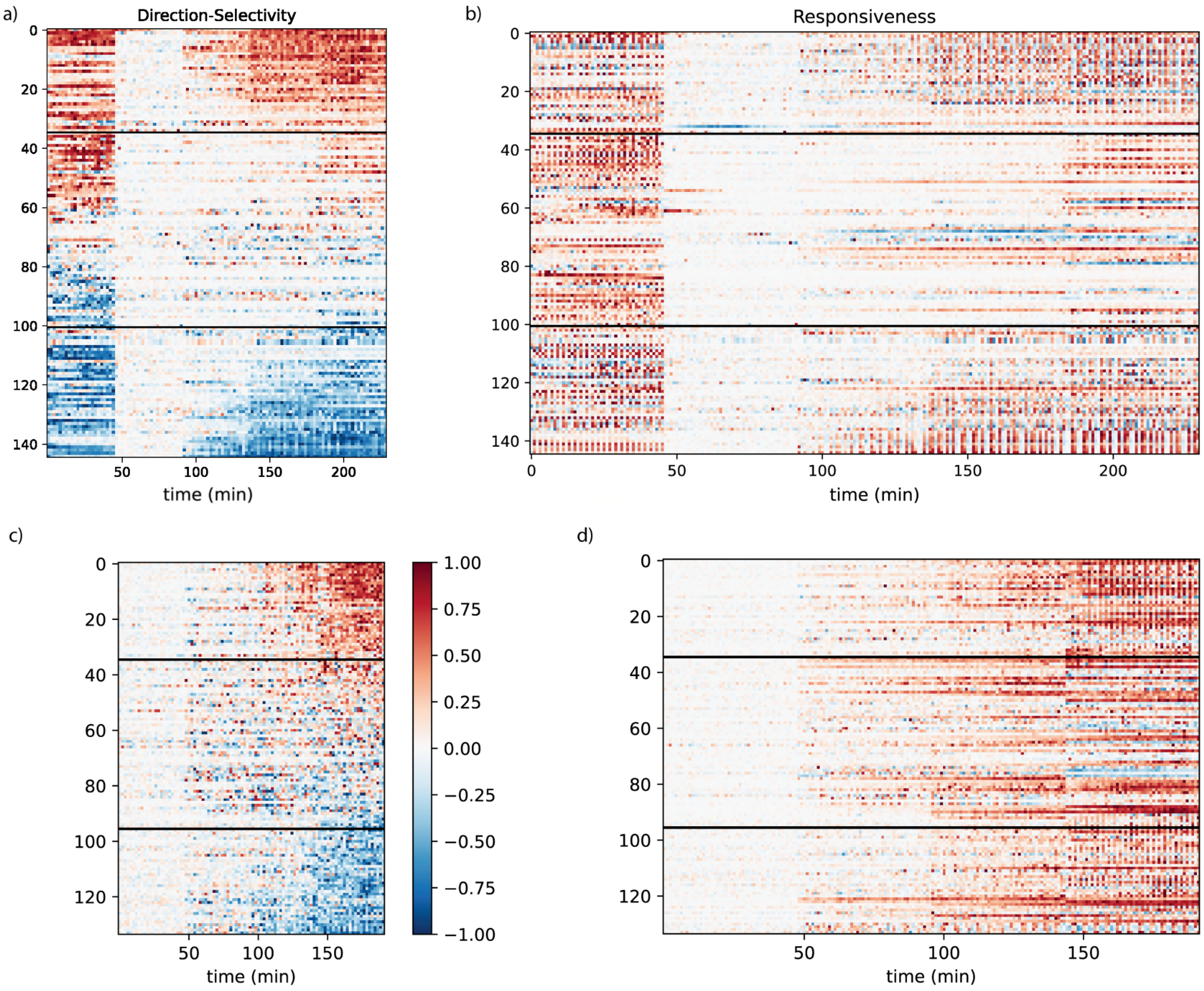

Supplemental Figure 13: Direction-Selective selective units emerge fully tuned. The responsiveness of neurons emerging from tricaine (b, one hour anesthesia, d, lifetime treatment), with units sorted according to the direction selectivity they exhibit (b according a, d according to c). This sorting aims to illustrate that direction selectivity emerges in lock step with responsiveness. Thus, the alternative hypothesis, that units first become active, then become tuned, is not supported.

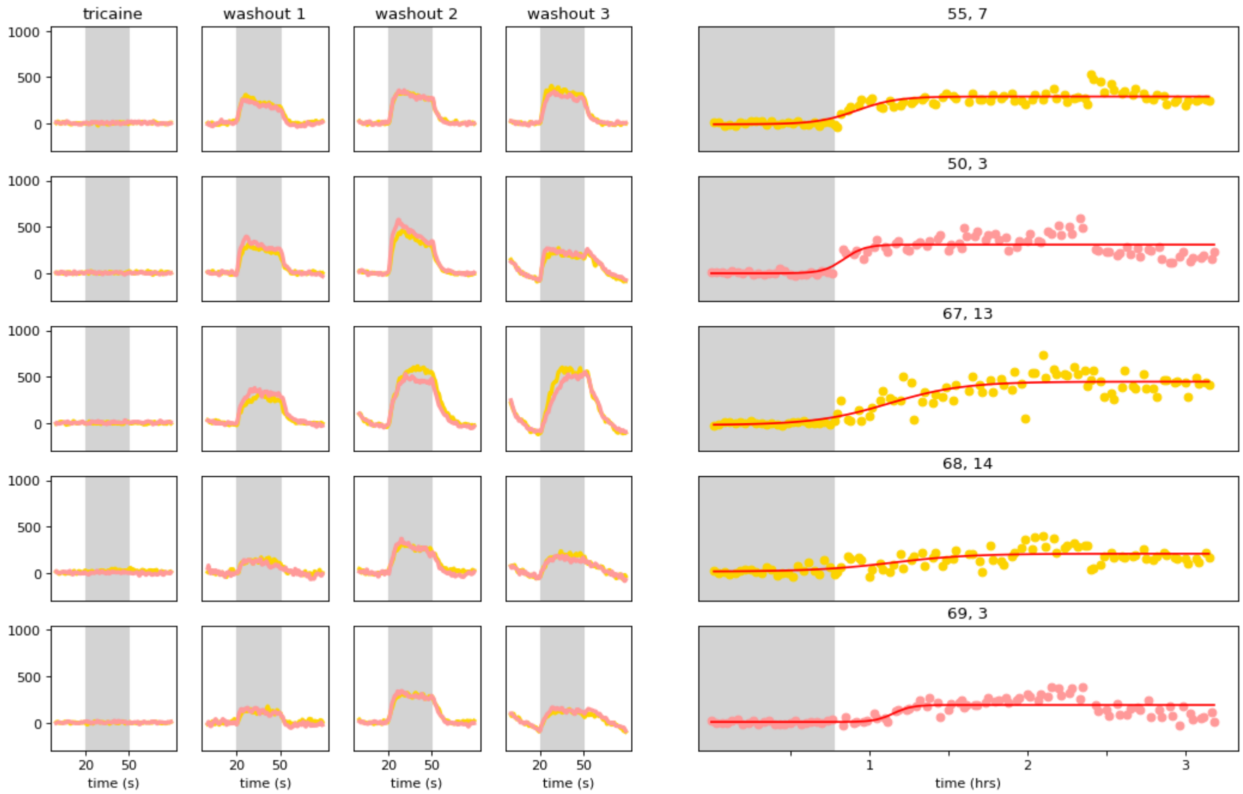

Supplemental Figure 14: Units that become active in the first hour after lifetime anesthesia washout.

Example units and fits from fish exposed to lifetime anesthesia. Square plots show the average response of the unit to leftward (pink) and rightward (yellow) motion averaged over an entire imaging session (akin to Figure 4f-h). Scatter plots show responsiveness-index of the preferred direction (indicated by color) of units in tectum, pretectum, and hindbrain (see Methods, Figure 2d, 4d), as well as a logistic fit to recovery. Scatter plots are titled by the midpoint (x_o) and time constant (k) of the logistic fit (plotted in Figure 4k-l).

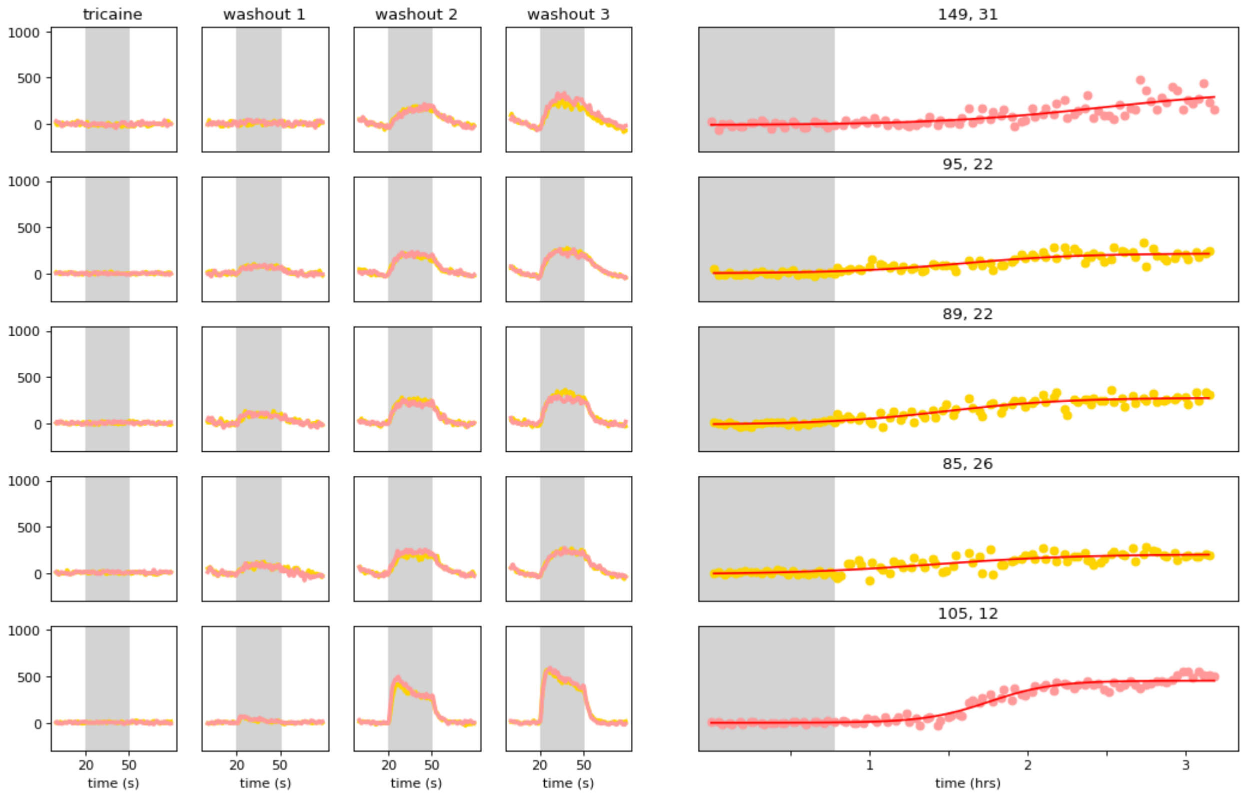

Supplemental Figure 15: Units that become active in the second hour after lifetime anesthesia washout.

Example units and fits from fish exposed to lifetime anesthesia. Square plots show the average response of the unit to leftward (pink) and rightward (yellow) motion averaged over an entire imaging session (akin to Figure 4f-h). Scatter plots show responsiveness-index of the preferred direction (indicated by color) of units in tectum, pretectum, and hindbrain (see Methods, Figure 2d, 4d), as well as a logistic fit to recovery. Scatter plots are titled by the midpoint (x_o) and time constant (k) of the logistic fit (plotted in Figure 4k-l).

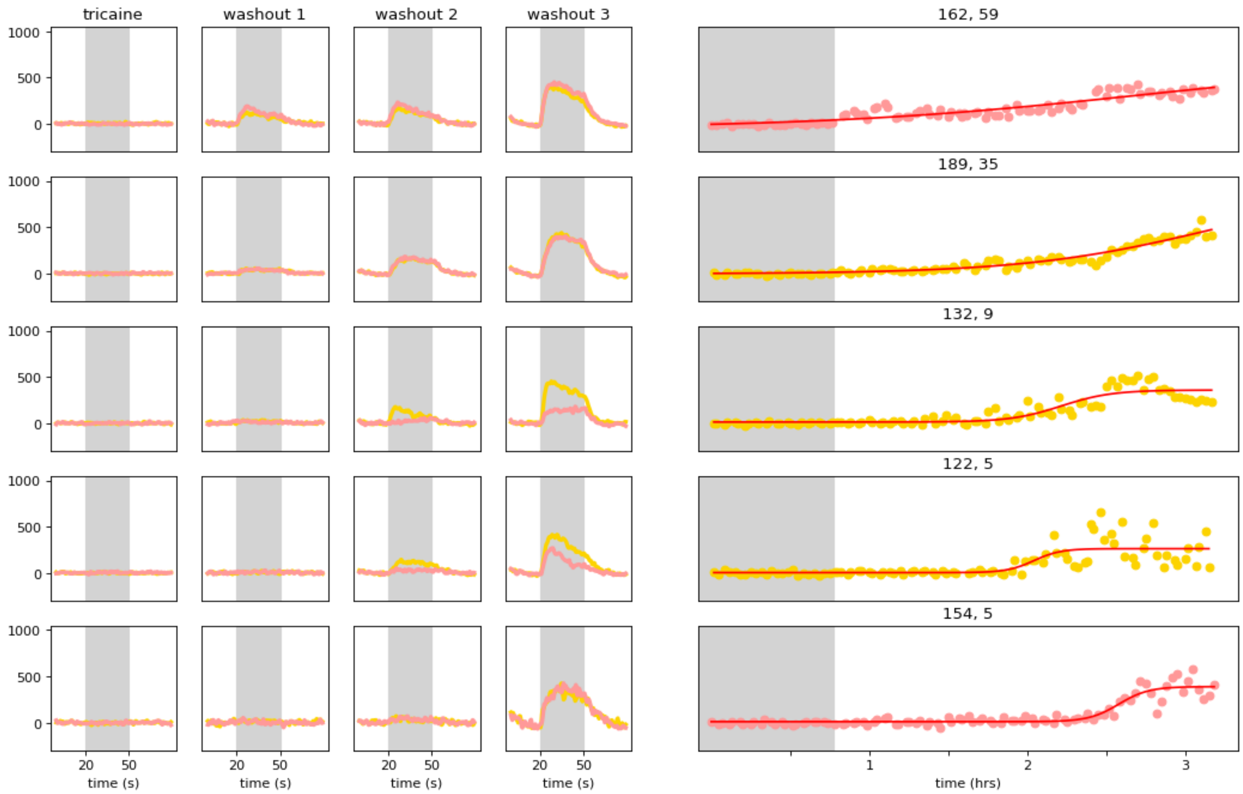

Supplemental Figure 16: Units that become active in the third hour after lifetime anesthesia washout.

Example units and fits from fish exposed to lifetime anesthesia. Square plots show the average response of the unit to leftward (pink) and rightward (yellow) motion averaged over an entire imaging session (akin to Figure 4f-h). Scatter plots show responsiveness-index of the preferred direction (indicated by color) of units in tectum, pretectum, and hindbrain (see Methods, Figure 2d, 4d), as well as a logistic fit to recovery. Scatter plots are titled by the midpoint (x_o) and time constant (k) of the logistic fit (plotted in Figure 4k-l).

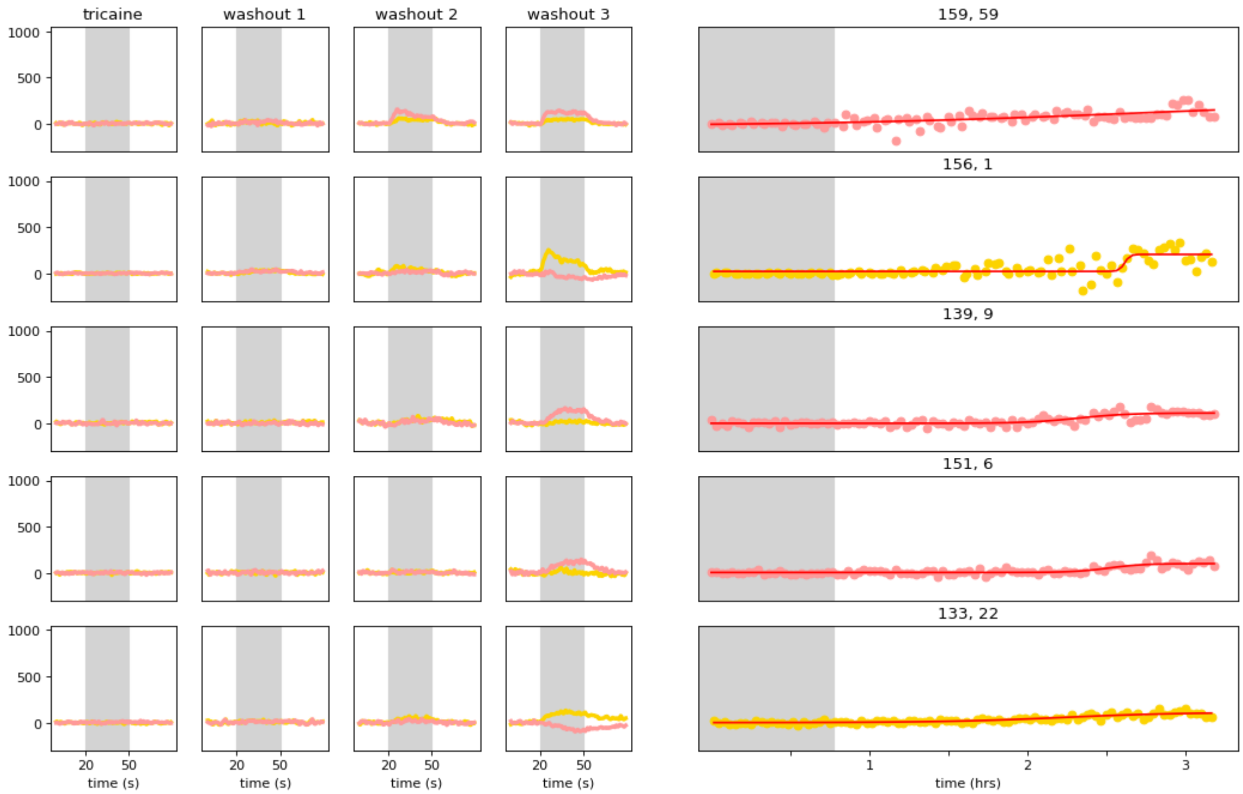

Supplemental Figure 17: Units that only weakly recover after lifetime anesthesia washout.
Example units and fits from fish exposed to lifetime anesthesia. Square plots show the average response of the unit to leftward (pink) and rightward (yellow) motion averaged over an entire imaging session (akin to Figure 4f-h). Scatter plots show responsiveness-index of the preferred direction (indicated by color) of units in tectum, pretectum, and hindbrain (see Methods, Figure 2d, 4d), as well as a logistic fit to recovery. Scatter plots are titled by the midpoint (x_0) and time constant (k) of the logistic fit (plotted in Figure 4k-l).

Supplemental Figures 18-19 (below): All remaining units not shown in Supplemental Figures 5-9 and 14-17.
Example units and fits from fish exposed to spot and lifetime anesthesia. Square plots show the average response of the unit to leftward (pink) and rightward (yellow) motion averaged over an entire imaging session (akin to Figure 2f-h, 4f-h). Scatter plots show responsiveness-index of the preferred direction (indicated by color) of units in tectum, pretectum, and hindbrain (see Methods, Figure 2d, 4d), as well as a logistic fit to recovery. Scatter plots are titled by the midpoint (x_0) and time constant (k) of the logistic fit (plotted in Figure 4k-l).

Supplementary Figure 18: Additional cells under temporary (1hr) tricaine.

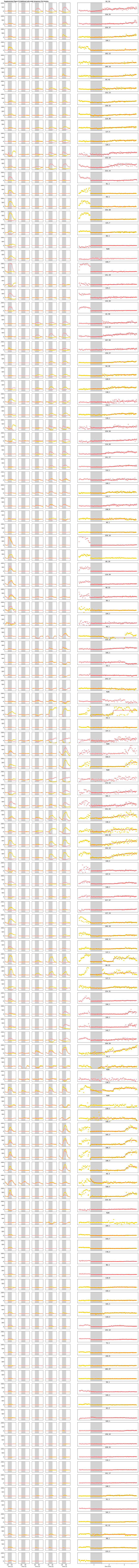

Supplementary Figure 19: Additional cells recovering from lifetime tricaine.

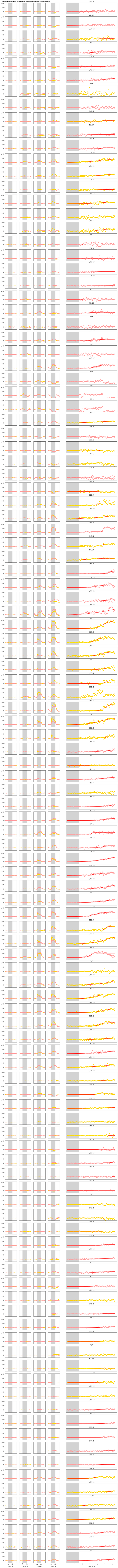

Supplement: Supplementary file 1 — Supplementary Information [file 41467_2023_44681_MOESM1_ESM.pdf]
